# A comparison of the population pharmacokinetics of rifampicin, isoniazid and pyrazinamide between hospitalized and non-hospitalized tuberculosis patients with or without HIV

**DOI:** 10.12688/wellcomeopenres.17660.1

**Published:** 2022-02-28

**Authors:** Noha Abdelgawad, Maxwell Chirehwa, Charlotte Schutz, David Barr, Amy Ward, Saskia Janssen, Rosie Burton, Robert J. Wilkinson, Muki Shey, Lubbe Wiesner, Helen McIlleron, Gary Maartens, Graeme Meintjes, Paolo Denti

**Affiliations:** 1 Division of Clinical Pharmacology, Department of Medicine, University of Cape Town, Observatory, 7925, South Africa; 2 Wellcome Centre for Infectious Diseases Research in Africa, Institute of Infectious Disease and Molecular Medicine, University of Cape Town, Observatory, 7925, South Africa; 3 Department of Medicine, University of Cape Town, Observatory, 7925, South Africa; 4 Wellcome Trust Liverpool Glasgow Centre for Global Health Research, University of Liverpool, Liverpool, L3 5QA, UK; 5 Amsterdam University Medical Centre, University of Amsterdam, Amsterdam, 19268, The Netherlands; 6 Khayelitsha Hospital, Department of Medicine, Khayelitsha, 7784, South Africa; 7 Department of Infectious Diseases, Imperial College London, London, W12 0NN, UK; 8 The Francis Crick Institute, London, NW1 1AT, UK

**Keywords:** Modelling & Simulation, Population pharmacokinetics, Tuberculosis, Hospitalization, TB/HIV

## Abstract

Background.

Early mortality among hospitalized HIV-associated tuberculosis (TB/HIV) patients is high despite treatment. The pharmacokinetics of rifampicin, isoniazid, and pyrazinamide were investigated in hospitalized TB/HIV patients and a cohort of outpatients with TB (with or without HIV) to determine whether drug exposures differed between groups.

Methods.

Standard first-line TB treatment was given daily as per national guidelines, which consisted of oral 4-drug fixed-dose combination tablets containing 150 mg rifampicin, 75 mg isoniazid, 400 mg pyrazinamide, and 275 mg ethambutol. Plasma samples were drawn on the 3rd day of treatment over eight hours post-dose. Rifampicin, isoniazid, and pyrazinamide in plasma were quantified and NONMEM ^®^ was used to analyze the data.

Results.

Data from 60 hospitalized patients (11 of whom died within 12 weeks of starting treatment) and 48 outpatients were available. Median (range) weight and age were 56 (35 - 88) kg, and 37 (19 - 77) years, respectively. Bioavailability and clearance of the three drugs were similar between TB/HIV hospitalized and TB outpatients. However, rifampicin’s absorption was slower in hospitalized patients than in outpatients; mean absorption time was 49.9% and 154% more in hospitalized survivors and hospitalized deaths, respectively, than in outpatients. Higher levels of conjugated bilirubin correlated with lower rifampicin clearance. Isoniazid’s clearance estimates were 25.5 L/h for fast metabolizers and 9.76 L/h for slow metabolizers. Pyrazinamide’s clearance was more variable among hospitalized patients. The variability in clearance among patients  was 1.70 and 3.56 times more for hospitalized survivors and hospitalized deaths, respectively, than outpatients.

Conclusion.

We showed that the pharmacokinetics of first-line TB drugs are not substantially different between hospitalized TB/HIV patients and TB (with or without HIV) outpatients. Hospitalized patients do not seem to be underexposed compared to their outpatient counterparts.

## Introduction

The mortality rate among treated hospitalized HIV-associated tuberculosis (TB/HIV) patients is high, ranging from 11% to 32% ^1, 2^. Hospitalized TB/HIV patients usually have some features of bacterial sepsis, with elevated venous lactate levels, and impaired intestinal barrier function, resulting in microbial translocation and high levels of circulating lipopolysaccharide, which mediates an inflammatory response ^3, 4^. Delayed gastric emptying and changes in gastric pH have been observed in severely ill patients ^5^. The gastrointestinal changes in severely ill patients could lead to differences in the rate and amount of drug absorption, and therefore affect drug exposure ^6^. Other changes in severely ill patients may include increased volume of distribution, changes in plasma protein binding, and changes in the intrinsic activity of drug metabolizing enzymes or in the hepatic blood flow that may affect drug clearance ^5– 7^. These changes could negatively affect the treatment outcome in vulnerable patients.

In addition to the extent of disease that could result in variable absorption, rifampicin’s extent and rate of absorption is highly variable ^8^. Rifampicin is mainly cleared by the liver and undergoes extensive first-pass metabolism ^9^. Saturable elimination has been reported for rifampicin at higher doses due to saturation of the biliary transport mechanisms ^9, 10^. After repeated administration, rifampicin exhibits autoinduction, in which it increases its own metabolism partly by activating the pregnane X receptor ^11^, which in turn induces the B-esterases in liver microsomes, which are responsible for the biotransformation of rifampicin to 25-desacetyl rifampicin ^12, 13^.

Isoniazid also has highly variable pharmacokinetics, mainly due to genetic polymorphism in N-acetyltransferase 2 (NAT2), the enzyme responsible for metabolizing the drug; the elimination of isoniazid in fast-acetylators is up to six times faster than the slow acetylators ^14^. Body composition parameters such as weight and fat-free mass (FFM) are usually good predictors of clearance and volume of distribution for many drugs. FFM is generally advised as a better scalar than bodyweight since it accounts for both the difference in body size and composition, unlike weight, which accounts for body size only ^15, 16^.

The aim of the study was to compare the pharmacokinetics of rifampicin, isoniazid, and pyrazinamide between hospitalized patients and outpatients recruited from the same hospital catchment area.

## Methods

### Study population

The study population is made up of two groups: the hospitalized patients and outpatients recruited as controls. The hospitalized study population for this pharmacokinetic (PK) sub-study was a subset from participants enrolled for an observational cohort study investigating the mortality causes in hospitalized TB/HIV patients carried out between November 2014 and November 2016 ^2^. Patients presenting to Khayelitsha Hospital in Cape Town, South Africa with TB/HIV who needed hospitalization and who survived to the third day of TB treatment were enrolled sequentially, as long as they still needed inpatient care and did not require transfer to a tertiary care facility for intensive care or investigations. The study team invited eligible hospitalized patients in the parent study to take part and discussed with them the study. Tuberculosis (TB) outpatients with or without HIV were recruited from around the same hospital catchment area as controls. The study team liaised with the clinic staff to ask any new patients when they were started on TB treatment if they would like to discuss taking part in the PK sub-study.

### Study design

All participants received a once daily dosing of antitubercular drugs that were given as 4-drug fixed-dose combination (FDC) tablets containing rifampicin-isoniazid-pyrazinamide-ethambutol at 150/75/400/275 mg, which were either Rifafour e-275 tablets (SANOFI) or Ritib tablets (PHARMACARE)). The number of tablets to be given to each participant is determined based on their weights according to the weight-based dosing of the South African national TB management guidelines outlined in Table 1. Clinical data and baseline blood tests were obtained at enrolment. The 12-week mortality outcome was documented for hospitalized patients.

**Table 1.** Summary of weight-based dosing.

Pre-treatment body weight (kg)	No. of RHZE FDC ^a^ (150/75/400/275 mg) during intensive treatment phase (daily dose for 2 months)
30–37	2
38–54	3
55–70	4
>70	5

^a^ RHZE, rifampin, isoniazid, pyrazinamide, and ethambutol fixed-dose combination tablets

### Ethics and consent

The study was approved by University of Cape Town Human Research Ethics Committee (UCT HREC reference: 057/2013) on 12 April 2013. All participants signed an informed consent form.

### Data collection

For each patient, age, sex, weight, height and details of concomitant medications were collected, and a complete medical history was recorded. Serum chemistry and a complete blood picture were carried out at the Groote Schuur National Health Laboratory Services on each participant on samples taken at enrolment for the PK study.

Participants were scheduled for a PK visit during their 3 ^rd^ day of treatment, when blood samples were drawn just before and 1, 2.5, 4, 6, and 8 hours after dose. Participants were required to fast overnight and they were given a standardized breakfast after the 1-hour sample and a standardized lunch between the 4- and 6-hour sample. Immediately following their collection, samples were put in an ice bath until being centrifuged in a cooling centrifuge and later stored at -80°C.

### Drug quantification

Plasma rifampicin, isoniazid and pyrazinamide concentrations were determined by validated liquid chromatography with tandem mass spectrometry assays at the Division of Clinical Pharmacology, University of Cape Town ^17^. The lower limit of quantification (LLOQ) was 0.117 mg/L for rifampicin, 0.105 mg/L for isoniazid, and 0.203 mg/L for pyrazinamide. The accuracy of the low-, medium-, and high-quality control samples ranged between 99.7% - 100.8% for rifampicin, 98.3% - 100.4% for isoniazid, and 88.1% and 92.3% for pyrazinamide. The precision of the quality control samples ranged from 4.7 – 7.7%, 3.0% - 5.1%, and 2.9% - 3.6% for rifampicin, isoniazid, and pyrazinamide, respectively ^2^.

### Pharmacokinetic and statistical analyses

A population pharmacokinetic model was developed for each of the three drugs using nonlinear mixed-effects modeling in NONMEM ^®^ version 7.4 and the algorithm first-order conditional estimation with eta-epsilon interaction (FOCEI) Pirana was used for model management, Perl-speaks-NONMEM (PsN) 4.9.0 was used for post-processing of NONMEM ^®^ results and and R version 3.6.2 was used for generating the figures ^18^. Different disposition models with first-order elimination were evaluated. The use of a lag time and transit compartments were tried to capture the delay in the first-order absorption process. Between-subject variability was evaluated for all disposition parameters and between-occasion variability was assessed for bioavailability, and other absorption parameters. Censored below the lower limit of quantification (BLQ) concentration values were handled as per Beal’s M6 method, in which the first BLQ values in series were replaced with LLOQ/2 and the subsequent BLQs were discarded ^19^. Residual unexplained variability was described using a combination of additive and proportional error components. The additive error was bound to be at least 20% of the LLOQ. Allometric scaling of clearance and volume parameters was tested as suggested by Anderson and Holford ^20^ using the fixed power exponents of 0.75 for clearance and 1 for volume. Body weight and FFM, calculated based on the formula in Janmahasatian *et al.* ^21^, were both tested for allometric scaling as body size descriptors. Since no NAT2 genotyping data were available, mixture modeling was used for the isoniazid pharmacokinetic model to distinguish between the clearances of different groups of metabolizers.

Following the development of a basic model, covariate testing was done. Various effects, including hospitalization, patient status (outpatients or hospitalized who survived or hospitalized who died within 12 weeks), drug formulation, and various biomarkers which indicate general organ dysfunction e.g. aspartate transaminase (AST), alanine transaminase (ALT), serum creatinine, serum urea, albumin, trefoil factor-3, and procalcitonin, were tested on clearance, bioavailability, and absorption parameters for all three drugs. 

The model development process and covariate inclusion were guided by physiological plausibility, model fit diagnostics including drop in the objective function value (OFV) and inspection of diagnostic plots. Comparison between nested models was done using the likelihood ratio test for the drop in OFV, assumed to be approximately χ ^2^ distributed with *n* degrees of freedom, where *n* is the number of additional estimated parameters. A *p*-value of 0.05 was generally used for inclusion and 0.01 for retention. Visual predictive checks (VPCs) were used to assess compatibility of the model with the observed data. Weakly-informative priors on the ratio of the volume of the central compartment (Vc) to the volume of the peripheral compartment (Vp), Vratio (Vc/Vp) with 30% uncertainty were used to stabilize the model for isoniazid PK. The typical values were obtained from a previously published model ^22^.

The precision of the parameter estimates, expressed as the 95% confidence intervals, was assessed by applying a nonparametric bootstrap with 500 iterations. A non-compartmental pharmacokinetic (PK) analysis of the same participants in this report has previously been published ^2^.

### Variability correlation across the three drugs

Correlations of unexplained variability in the pharmacokinetic parameters: clearance, bioavailability, area under the curve from time 0 to 24 hours (AUC _0-24h_) and absorption between each of the three drugs were assessed to check if there was any relation between the unexplained variabilities in each pharmacokinetic parameter between the three drugs. There were two occasions per patient. An occasion is defined as any dosing event followed by at least one sample. When checking the correlation between variabilities, only the variability from the primary occasion was included i.e. the occasion associated with the predose was excluded.

## Results

### Study data

A total of 108 patients completed the study; 60 were hospitalized TB/HIV patients, and 48 were TB outpatients, of whom 29 were HIV-positive. Table 2 provides a summary of the participants characteristics. Four hospitalized patients (n=4, 3.7%) had missing height values, which were replaced by the regression-imputed values based on sex. Two hospitalized patients with renal impairment had individual tablets for each drug instead of the FDC to allow adjustment of the ethambutol dose, and one hospitalized patient had the tablets crushed, mixed with water, then inserted via a nasogastric tube. All patients had blood samples collected on the 3 ^rd^ day of treatment, except for one participant, in whom it was found out during the study that there was an earlier dose, so the collected samples were on the 4 ^th^ day of treatment. 

**Table 2.** Participants baseline characteristics.

	Median (interquartile range) or no. (%) of participants given		
	Hospitalized (n = 60)	Outpatients (n = 48)	Total (n = 108)
No. (%) of:			
Females	31 (52%)	12 (25%)	43 (40%)
Age (yr)	38 (32 – 41)	36 (32 – 42)	37 (32 – 41)
Weight (kg)	55 (48 – 60)	58 (52 – 63)	56 (49 – 62)
Fat-free mass (kg)	40 (36 – 47)	47 (41 – 51)	43 (38 – 49)
No. (%) of:			
HIV-positive	60 (100%)	29 (60%)	89 (82%)
Total bilirubin (μmol/L) ^a^	10.0 (6.00 – 14.5)	10.0 (7.00 – 14.0)	10.0 (6.00 – 14.0)
Conjugated bilirubin (μmol/L) ^b^	6.00 (3.00 – 9.00)	6.00 (4.00 – 8.00)	6.00 (3.00 – 9.00)
Lactate (mmol/L) ^c^	1.55 (1.13 – 2.30)	1.60 (1.20 – 1.95)	1.60 (1.20 – 2.00)
Aspartate aminotransferase, AST (U/L) ^d^	50.0 (34.0 – 78.9)	30.0 (21.0 – 50.0)	38.0 (27.0 – 69.0)
Alanine aminotransferase, ALT (U/L) ^e^	27.0 (18.0 – 47.0)	20.0 (13.5 – 30.0)	24.0 (16.0 – 40.8)

^a^ Total bilirubin was missing for 1 hospitalized patient and 1 outpatient ^b^ Conjugated bilirubin was missing for 5 hospitalized patients and 3 outpatients ^c^ Lactate was missing for 2 hospitalized patients and 13 outpatients ^d^ AST was missing for 8 hospitalized patients and 3 outpatients ^e^ ALT was missing for 1 hospitalized patient and 1 outpatient

A total of 108 pharmacokinetic profiles with 632 concentration-time observations for each of the three drugs were available for analysis. The number of observations that were BLQ were 33 (5.2%), 88 (13.9%), and 1 (0.2%) for rifampicin, isoniazid, and pyrazinamide, respectively, most of which were predose samples. The 12-week mortality rate of hospitalized patients was 11/60 (18%). One participant was lost to follow up after 2 months; the participant’s results were included in the survived group. We chose to stratify the analysis of the hospitalized patients into those who survived and those who died within 12 weeks as an indicator of severity of the patients’ sickness.

### Rifampicin pharmacokinetics

**Figure 1.**
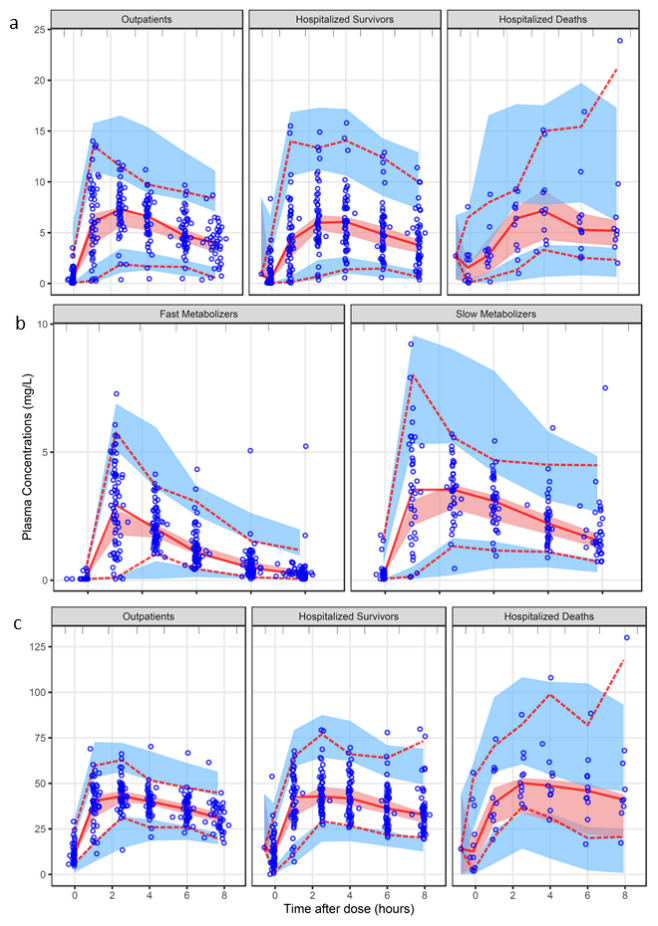
Visual predictive check (VPC) (n=1000) showing plasma drug concentration versus time after dose for the final models of each drug: **a**) for rifampicin stratified by patient group; **b**) for isoniazid stratified by metabolizer status; **c**) for pyrazinamide stratified by patient group. The circles are the original observations; the solid line and the dashed lines are the median, 5 ^th^ and 95 ^th^ percentiles of the observed data; the shaded areas are the 95% confidence intervals of the same percentiles as simulated by the model. A suitably fitting model will have most of the observed percentiles within the simulated confidence intervals.

Rifampicin pharmacokinetics was best characterized by a one-compartment disposition model with first-order elimination, and absorption described by a chain of transit compartments. The parameter values of the final model are shown in Table 3. The model fit the data well as shown in the VPC in Figure 1. Apparent clearance (CL) and apparent volume of distribution (V) were allometrically scaled using FFM as a body size descriptor. Allometric scaling by FFM (difference in OFV, dOFV = -30, df = 2, *p*-value = <0.001) resulted in a more significant drop than scaling by total body weight (dOFV = -7.7, df = 2, *p*-value = 0.02). The typical CL and V values for a participant with the median FFM were 8.82 L/h and 56.8 L, respectively. The final parameter estimates are shown in Table 3.

**Table 3.** Final population pharmacokinetic parameter estimates for rifampicin, isoniazid and pyrazinamide.

Parameter	Typical value (95% CI) ^a^		
	Rifampicin	Isoniazid	Pyrazinamide
Clearance (L/h) ^b^	8.82 (8.10 – 9.48)	-	2.61 (2.48 – 2.75)
Clearance of Fast metabolizers (L/h)	-	25.5 (22.7 – 28.7)	-
Clearance of Slow metabolizers (L/h)	-	9.76 (8.28 – 11.2)	-
Proportion of fast NAT2 metabolizers (%)	-	64.5 (54.4% – 75.8%)	-
Volume of distribution (L) ^b^	56.8 (53.9 – 61.2)	59.0 (54.7 – 64.2)	36.0 (34.4 – 37.9)
Intercompartmental clearance, Q (L/h)	-	1.43 (0.874 – 2.14)	-
Peripheral volume, Vp (L) ^c^	-	30.7 (25.9 – 37.1)	-
Absorption rate constant, ka (h ^-1^)	1.38 (1.04 – 1.70)	2.43 (1.80 – 6.50)	1.92 (1.53 – 2.59)
Mean transit time, MTT (h)	0.342 (0.259 – 0.534)	0.442 (0.266 – 0.781)	0.379 (0.220 – 0.566)
No. of absorption transit compartments (.)	12 fixed	8 fixed	4 fixed
Bioavailability, F (%)	100 fixed	100 fixed	100 fixed
% difference in mean absorption time (MAT) for hospitalized survivors ^d^	+49.9% (+2.80% – +80.9%)	-	-
% difference in MAT for hospitalized deaths ^d^	+154% (+63.9% – 351%)	-	-
Exponent of power relationship between Clearance and conjugated bilirubin ^e^	-0.333 (-0.474 – -0.194)	-	-
Between-subject variability for clearance (BSVCL) (%)	42.4 (37.3 – 49.4)	25.3 (17.2 – 33.4)	19.9 (13.2 – 25.0)
Fold change in BSVCL for hospitalized survivors ^f^	-	-	1.70 (1.26 – 2.65)
Fold change in BSVCL for hospitalized deaths ^f^	-	-	3.56 (1.64 – 6.53)
Between-occasion variability (BOV) (%) for:			
Bioavailability	21.3 (16.4 – 27.6)	34.9 (26.6 – 40.3)	10.5 (4.13 – 15.1)
Absorption rate constant, ka	119 (100 – 137)	122 (84.0 - 186)	75.3 (40.3 – 95.3)
Mean transit time, MTT	93.8 (67.4 – 111)	99.7 (45.9 – 172)	102 (61.9 – 145)
Proportional error (%)	17.2 (14.9 – 18.5)	13.9 (12.0 – 16.4)	11.4 (7.59– 13.9)
Additive error (mg/L)	0.0234 fixed ^g^	0.021 fixed ^g^	2.48 (1.47 – 3.44)

^a^ Values in parentheses are empirical 95% confidence intervals, obtained with a 500-sample nonparametric bootstrap ^b^ The values of CL/F and V/F were allometrically scaled, so the typical values reported here refer to the median body weight of 66 kg and the median FFM of 43 kg of the cohort included in the model. ^c^ The peripheral volume, Vp, was calculated from the estimated Vratio (Vc/Vp) and Vc. A prior of 2.02 was included on Vratio with 30% uncertainity. ^d^ Patient status effect was modeled on ka and 1/MTT simultaneously using the same effect parameter, theta (θ). ka for hospitalized = TVka × θ; MTT for hospitalized = TVMTT / θ. Mean absorption time = MTT+1/ka calculated for each group was 1.1 h for outpatients, 1.6 h for hospitalized survivors, and 3.2 h for hospitalized deaths. ^e^ *BRC Effect on CL* = ( *BRC/median BRC*) ^-0.326^; *CL = TVCL x BRC Effect on CL* ^f^ BSVCL for hospitalized = BSVCL × fold change. i.e. BSVCL is 33.8% for hospitalized survivors and 70.8% for hospitalized deaths. ^g^ The estimate of the additive component of the error was not significantly different from its lower boundary of 20% of LLOQ, so it was fixed to this value.

No difference in CL or bioavailability was found between hospitalized patients and outpatients. Nonetheless hospitalized patients, and even more so those who died in the first 12 weeks, were found to absorb rifampicin slower than outpatients (dOFV = -16.1, df = 2, *p*-value = <0.001). The effect of patient group (outpatients, hospitalized survivors and hospitalized deaths) was modeled on the absorption rate constant (ka) and 1/mean absorption time (MTT) simultaneously using the same effect parameter, theta (θ), as outlined in the formulae below.


$$MTT_{group}=\frac{MTT_{outpatients}}{{\text{θ}}_{patient\;group\;effect}}\;k_{a_{group}}={\text{θ}}_{patient\;group\;effect}\cdot k_{a_{outpatients}}$$

Where *MTT _outpatients_* is the typical mean transit time for the outpatients in hours, *MTT _group_* is the mean transit time for hospitalized survived or hospitalized deaths group in hours, *k _a _group__* is the absorption rate constant for hospitalized survivors or hospitalized deaths group in hour ^-1^, and *k _a _outpatients__* is the typical absorption rate constant for outpatients in hour ^-1^

On average, hospitalized patients who survived had a mean absorption time (MAT) of 1.6 h (accounting for both MTT and ka), while the value was 2.7 h for hospitalized patients who died in the first 12 weeks, compared to 1.1 h for outpatients.

Additionally, we found that higher values of conjugated bilirubin (BRC) were correlated with lower values of rifampicin CL (dOFV = -17.3, df = 1, *p*-value = <0.001), according to the power relationship outlined below


$$CL_{i}=CL_{typical}\cdot {\left(\frac{BRC_{i}}{BRC_{median}}\right)}^{\beta_{BRC}}$$

**Figure 2.**
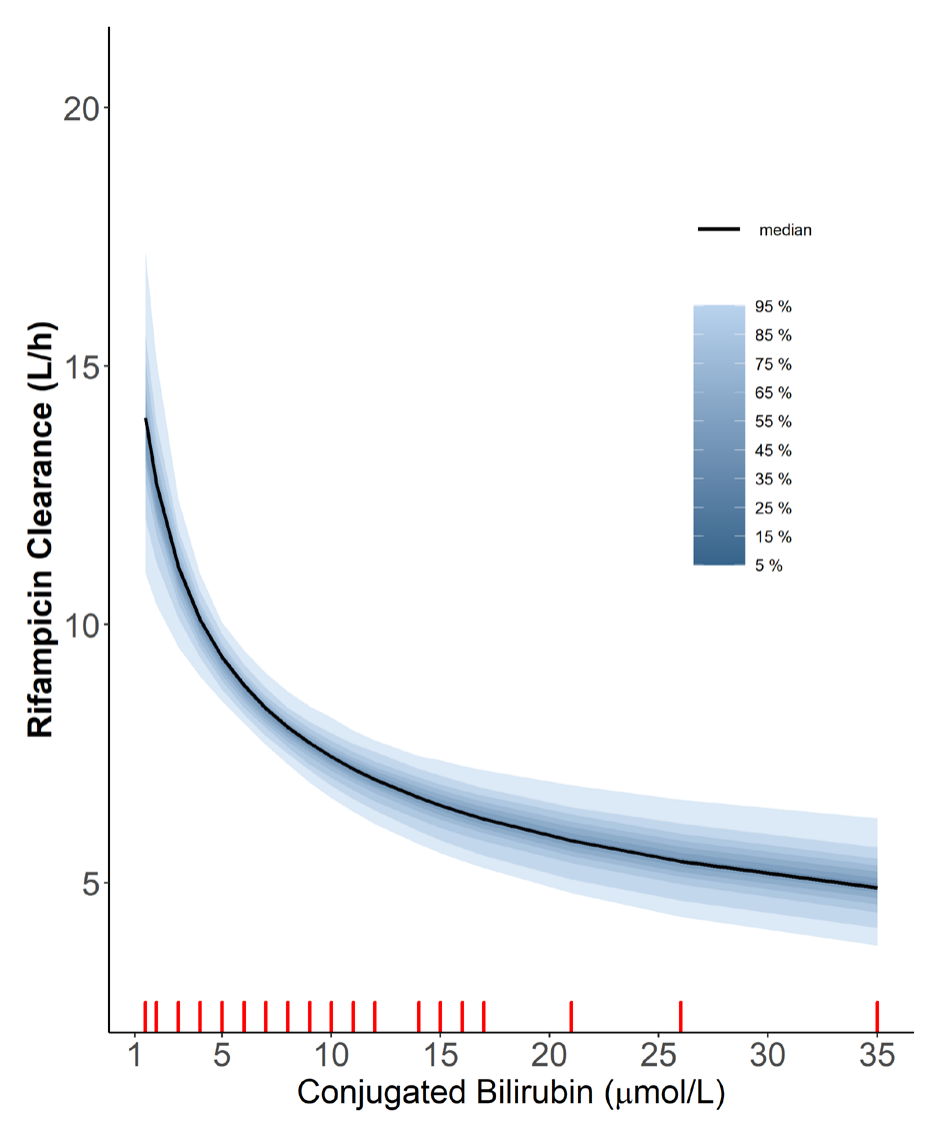
The relationship of rifampicin clearance vs the level of conjugated bilirubin using the power function. The red ticks represent the values of conjugated bilirubin observed in our cohort. The solid line represents the median and the shaded areas represent the uncertainty distribution around the effect due to the precision of parameter estimates.

Where *CL _i_* is the clearance for patient *i*, *CL _typical_* is the typical clearance, which is 8.82 L/h, *BRC _i_* is the BRC for patient *i*, *BRC _median_* is the BRC median in all patients (6 µmol/L) and *β _BRC_* is the exponent of power relationship between CL and BRC, estimated to be -0.333. The power function was a better fit for the relationship between BRC and CL compared to linear, piece-wise linear. The relationship is depicted in Figure 2. Both total bilirubin (BRT) and BRC were found to correlate significantly with CL; however, the two covariates (i.e. BRT and BRC) are highly positively correlated (r = 0.860), so only one of them was included in the final model. BRC was chosen over BRT because it resulted in a more significant drop in OFV. We tried incorporating saturation of elimination of rifampicin and the first-pass metabolism into the model. However, both models resulted in a marginal improvement of the fit. Therefore, we decided to keep the model more straightforward and not include either saturation or first-pass metabolism in the final model.

None of the biomarkers tested were found to correlate significantly to CL, except for the level of venous lactate and AST. However, the correlation between clearance and lactate or AST was less significant than the correlation with BRC, so only the effect of BRC was included in the final model.

An effect for the formulation was found to be statistically significant (dOFV = -12.9, df =1, p-value < 0.001), with the individual tablets having 21.8% of the bioavailability of FDC. However, only two participants were on individual tablets (n = 2, 1.85%) instead of the FDC, one of whom vomited during the study.

### Isoniazid pharmacokinetics

A two-compartment disposition model with first-order absorption with a chain of transit compartments and first-order elimination proved to fit the data best. The final parameter estimates are shown in Table 3.

A 2-compartment model was a better fit than the 1-compartment in terms of a significant drop in OFV, which was about 42 points, and by a VPC, but the model was unstable and Vp could not be reliably estimated. To stabilize the estimate of the Vp, a prior was included on the Vratio (Vc/Vp) with a value of 3.728 with 30% uncertainty ^22^. Allometric scaling of CL and Vc using FFM was used because it caused a more significant drop in the OFV of 24.9 points instead of weight which caused a drop of only 15.5 points.

Mixture modeling was used to account for the differences in CL between fast and slow metabolizers in place of NAT2 genotype testing (dOFV = -15.5, df =2, p-value < 0.0005). The proportion of fast metabolizers was estimated to be 64.5%. The typical clearance values were estimated to be 25.5 L/h and 9.76 L/h for fast and slow metabolizers. A three-component mixture distinguishing into fast, intermediate, and slow metabolizers was examined but was not supported by the data. Figure 1 includes a VPC for the final isoniazid model stratified by metabolizer type, indicating that the model fit the data well. Isoniazid pharmacokinetics were not different in hospitalized patients compared to outpatients.

### Pyrazinamide pharmacokinetics

A one-compartment disposition model with first-order elimination and first-order absorption with transit absorption compartments best fit the data. Allometry with FFM was applied to CL and V (dOFV = - 32.6 points for FFM, better than total body weight, dOFV = -28.3). Final parameter values are displayed in Table 3.

No significant differences were found in the CL, bioavailability, or absorption between hospitalized and outpatients. The between-subject variability in CL was significantly higher among hospitalized patients, i.e. 20% for outpatients vs 33.8% for hospitalized patients who survived vs 70.8% for hospitalized patients who died within 12 weeks (dOFV = -27, df=2, *p*-value < 0.001). A VPC showing that the model correctly captures the data for pyrazinamide is shown in Figure 1.

Neither the HIV status nor the CD4+ cell count influenced the pharmacokinetics of any of the three drugs. The effect of efavirenz co-administration (n = 9) was tested on the CL and bioavailability of all three drugs. No significant effect for the co-administration of efavirenz was found.

### Variability correlation across the three drugs

The correlations of the remaining unexplained variability in clearance, bioavailability, AUC _0-24h _and absorption among the three drugs were assessed and the results are shown in the correlation matrix in Figure 4. The equations used to calculated the unexplained variabilities for each parameter are shown below Figure 4. Moderate correlations were found for all, except for absorption, which ranged between 68.4% - 84.6%.

## Discussion

The main finding of our analysis is that the overall drug exposures for rifampicin, isoniazid, and pyrazinamide are similar between hospitalized TB/HIV patients and TB outpatients. For rifampicin, our model showed that absorption was slower in hospitalized patients, even slower among hospitalized patients who died within 12 weeks, and that higher levels of bilirubin were associated with lower rifampicin clearance. For pyrazinamide, the between-subject variability in CL was higher among hospitalized patients, and higher among hospitalized patients who died compared to hospitalized patients who survived.

There are limited data comparing the pharmacokinetics of first-line anti-TB drugs between hospitalized patients and outpatients. A non-compartmental analysis (NCA) of the data from this pharmacokinetic study was published by Schutz *et al.* ^2^. The NCA show that the overall exposures of all three drugs among hospitalized patients and outpatients were similar, which is in line with our findings. The pharmacokinetic parameters from our models for rifampicin, isoniazid and pyrazinamide were comparable to those from other similar studies ^8, 23– 26^

### Rifampicin PK model

**Figure 3.**
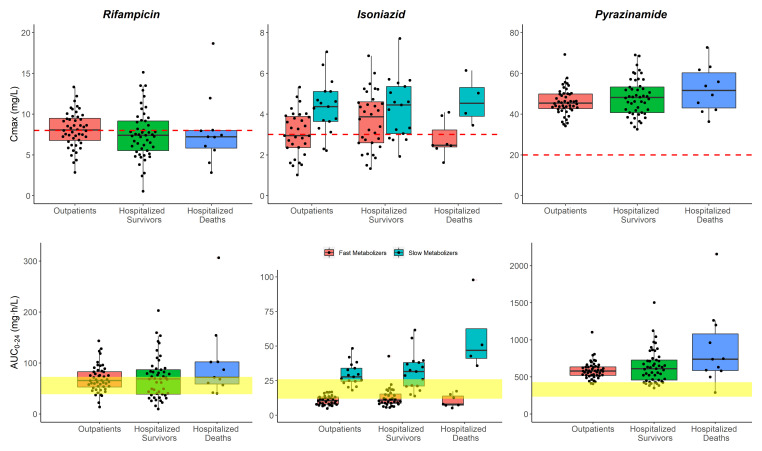
Box and whisker plots of the model-derived individual C _max_ and AUC _0-24_ for the three drugs. The dots are individual values, and the whiskers represent the 2.5 ^th^ and 97.5 ^th^ percentiles. The dashed line represents the currently recommended minimum threshold: 8 mg/L for rifampicin, 3 mg/L for isoniazid, and 20 mg/L for pyrazinamide. The yellow shaded areas represent the exposure targets based on Stott *et al.* for rifampicin ^38^ and Daskapan *et al.* for isoniazid and pyrazinamide ^37^. This is only for visualization purposes; no statistical tests can be carried out here since dose amounts are not accounted for.

The structural model we developed for rifampicin was similar to previously developed rifampicin models ^8^. However, CL values are lower in this analysis because sampling was done on the third day of treatment, where autoinduction is still not significant. Regarding the differences in absorption, published articles report that critically ill patients tend to have a more impaired absorption of drugs through a decreased barrier gut function and delayed gastric emptying, which lead to reduced perfusion of the gastrointestinal tract ^5– 7^. We reason that only rifampicin’s absorption out of the three drugs was affected by these gastrointestinal changes because of rifampicin’s low solubility ^27^, whereas both isoniazid and pyrazinamide have high solubility according to the biopharmaceutics classification system ^28, 29^. Rifampicin’s absorption is mainly from the stomach and proximal intestine ^30^ and is more likely to be easily affected by changes in the gastric pH ^9^. As a result, the Cmax for hospitalized patients tends to be lower than that of the outpatients, while the AUC _0-24h_ does not seem to be affected as shown in Figure 3.

**Figure 4.**
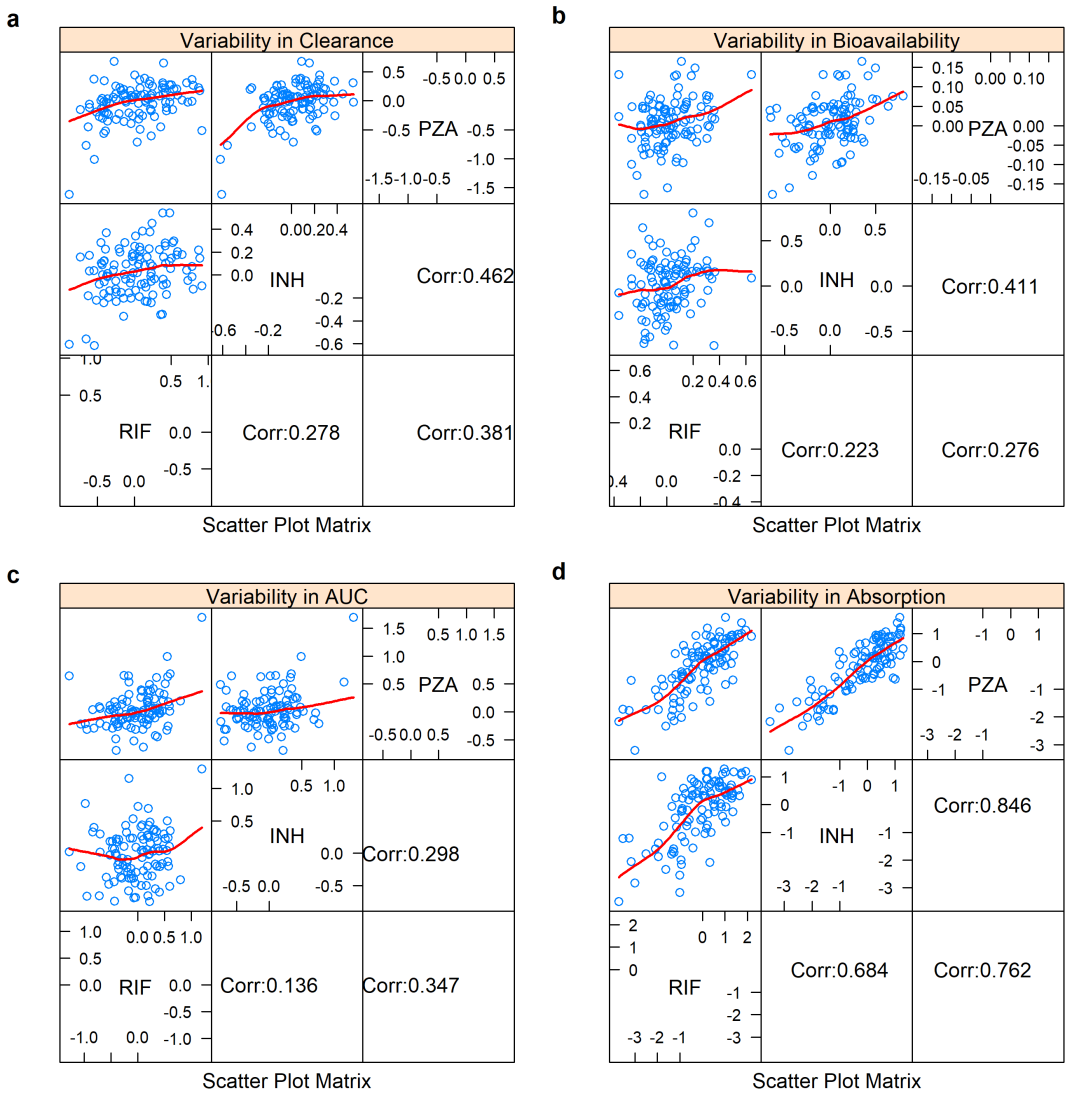
Correlation matrix for the unexplained variability in **a**) clearance, **b**) bioavailability, **c**) area under the curve (AUC _0-24h_), and **d**) absorption between the three drugs. The correlation coeffeicient is shown in the lower panel. Only the variability from the main occasion was included (not the predose). Variability in clearance = BSVCL + BOVCL. Variability in bioavailability = BSVBIO + BOVBIO. Variability in AUC = BSVBIO + BOVBIO - BSVCL. Variability in absorption = BSVKA + BOVKA - BSVMTT - BOVMTT.

Rifampicin and its major metabolite are mostly excreted through the biliary tract, the same tract that excretes bilirubin. Therefore, higher bilirubin levels correlate with lower rifampicin clearances since bilirubin and rifampicin compete for the same elimination pathway ^9, 31^.

While marked differences have been reported in the rate and extent of absorption with different formulations ^32^, only two patients in our study were on individual tablets, and one of them vomited during the study, therefore the effect of formulation was not included in the final model. Saturation of clearance and first-pass metabolism have been reported previously for rifampicin ^33^. While there was no significant effect for either HIV status or efavirenz co-administration, previous studies have reached contrasting results regarding both. Some studies found no significant difference in rifampicin concentrations ^34^, while others found decreased rifampicin levels in HIV-positive TB patients ^35– 37^. Nevertheless, a meta analysis by Stott *et al.* ^38^ concluded that HIV positivity had no effect on rifampicin exposure.

### Isoniazid PK model

The estimated proportion of fast acetylators/metabolizers of 64.5% is in line with the proportion of fast/intermediate acetylators in South Africans from previous publications which ranges between 48% - 60% ^39– 41^. In previously published pharmacokinetic studies in adults, isoniazid’s CL ranged between 22 and 26 L/h in fast metabolizers and between 10 and 16 L/h in slow metabolizers, which are similar to this study’s results ^23, 24^.

We opted for adding a prior on the ratio of the two volumes (Vc/Vp) instead of the Vp because this is expected to be more consistent across studies which may be characterized by different body size and/or differences in bioavailability.

Inadequate exposure of isoniazid has been observed in fast metabolizers across the three patient groups as shown in Figure 3; the AUC _0-24h_ levels on average were below the recommended targets. This effect has been previously reported by Sundell *et al.* ^42^.

### Pyrazinamide PK model

The values reported for the pyrazinamide model are in line with the values from previously published models. While there were no significant differences in pharmacokinetic parameters between the patient groups, we found a difference in the between-subject variability in CL (BSV-CL). The BSV-CL in outpatients was 19.9, 33.8% among hospitalized patients who survived and 70.8% among hospitalized patients who died. The differences in variability could be explained by the severity of the illness of the different patient groups. More critically sick patients have factors such as degree of hepatic impairment, sepsis that may lead to more variability.

### Variability correlation across the three drugs

There was no strong correlation between the unexplained variability in clearance, AUC _0-24h_, and bioavailability across the three drugs. The moderate correlation in the unexplained variability in absorption could be explained by the fact that most of the participants were taking an FDC formulation. Therefore, the factors affecting the tablet distintegration and dissolution e.g. manufacturing variables, and drug absorption, e.g., gastrointestinal contents, will be the same across the three drugs in any particular patient. 

One limitation of the study is that NAT2 genotype testing was not carried out for the participants’ samples, but this was resolved by using a mixture model to assign each participant to either being a fast or a slow metabolizer. Another limitation is that blood samples were collected on the 3 ^rd^ day (4 ^th^ day for one hospitalized participant) of treatment, which did not allow for the inclusion of autoinduction of rifampicin’s clearance in the model. However, hospitalized patients are at substantial risk of death within 7 days of admission before autoinduction is established, so the exposures we report here are relevant for these patients.

In summary, no important differences in any of the exposures of the three drugs: rifampicin, isoniazid, and pyrazinamide between hospitalized TB/HIV patients and TB outpatients were observed. The main findings of the analysis were that rifampicin’s absorption is slower in hospitalized patients (and slower in hospitalized patients who died compared to those who survived) and that patients with higher levels of bilirubin had lower rifampicin clearance. Pyrazinamide’s clearance was more variable among hospitalized patients (and more variable in hospitalized patients who died compared to those who survived).

## Data availability

### Underlying data

The data that support this research cannot be adequately de-identified in accordance with the Safe Harbor method since the dataset contains full treatment dates. The data including the drug concentrations, dosing and sampling dates and times, plus the covariates tested can be made available upon reasonable request to *bona fide* researchers by contacting Paolo Denti ( paolo.denti@uct.ac.za)
