# Supplementary material for: Pharmacokinetics of antitubercular drugs in patients hospitalized with HIV-associated tuberculosis: a population modeling analysis
Source: Wellcome Open Res. 2024 Mar 1;7:72. Originally published 2022 Feb 28. [Version 3] doi: 10.12688/wellcomeopenres.17660.3 (PMC10050909; doi:10.12688/wellcomeopenres.17660.3)
Supplement: Peer review history

# Reviewer report: Reviewer response for version 3

**DOI:** 10.21956/wellcomeopenres.23151.r75826

**Published:** 2024-03-07

**Authors:** Fajri Gafar

**Affiliations:** 1 Unit of PharmacoTherapy, -Epidemiology and -Economics, Groningen Research Institute of Pharmacy, University of Groningen, Groningen, The Netherlands

Thank you for the revised version of the manuscript. The reviewer has no further comments to make.

Is the work clearly and accurately presented and does it cite the current literature?

Partly

If applicable, is the statistical analysis and its interpretation appropriate?

Yes

Are all the source data underlying the results available to ensure full reproducibility?

Yes

Is the study design appropriate and is the work technically sound?

Yes

Are the conclusions drawn adequately supported by the results?

Yes

Are sufficient details of methods and analysis provided to allow replication by others?

Yes

Reviewer Expertise:

Clinical pharmacology of anti-TB drugs, pharmacotherapy of TB, epidemiology of TB.

I confirm that I have read this submission and believe that I have an appropriate level of expertise to confirm that it is of an acceptable scientific standard.

# Reviewer report: Reviewer response for version 2

**DOI:** 10.21956/wellcomeopenres.20580.r55569

**Published:** 2023-03-28

**Authors:** Fajri Gafar

**Affiliations:** 1 Unit of PharmacoTherapy, -Epidemiology and -Economics, Groningen Research Institute of Pharmacy, University of Groningen, Groningen, The Netherlands

I read with interest the manuscript “Pharmacokinetics of antitubercular drugs in patients hospitalized with HIV-associated tuberculosis: a population modeling analysis” submitted by Noha Abdelgawad and colleagues to Wellcome Open Research.

 This is an interesting study and I commend the authors for their great work. The methods, particularly the pharmacokinetic and statistical analyses, are well described. The results are presented clearly and are easy to follow and understand. Aiming to increase the scope of the manuscript and its applicability, the reviewer hopes that the authors can address the following few minor comments and suggestions:

 1) In the introduction, the authors mentioned that the aim of the study was to compare the pharmacokinetics of anti-TB drugs between hospitalized patients and outpatients, but it turned out that the hospitalized patients in this study only included those with HIV-associated TB, although the hospitalized patients could also be those with other severe forms of disease like TB meningitis and miliary TB; please clarify.

 2) It is not clear from the introduction what the hypothesis of the study is. What do the authors expect from comparing the pharmacokinetics of anti-TB drugs in hospitalized patients and outpatients with TB (e.g., lower drug exposures in hospitalized patients)? A brief description of the hypothesis will help readers understand the study.

 3) Regarding the study population, the authors mentioned that patients with HIV-associated TB who were too ill to receive initial treatment as outpatients were included as hospitalized patients. However, it is not clear how ill the patient is to be eligible for hospitalization. The authors should briefly describe the criteria for hospitalization in these patients with HIV-associated TB (e.g., based on WHO clinical staging of HIV/AIDS, CD4 count, viral load, and/or other clinical parameters). Adding details of these criteria in Table 2 is also a good option to help readers understand the characteristics of the study population.

 4) The authors mentioned that outpatients with or without HIV-associated TB were recruited from the same hospital catchment area as controls. However, it is not clear from the text why outpatients with and without HIV were included as controls while only inpatients with HIV were included as cases. It is also unclear whether these outpatients were recruited during the same period as the inpatients (2014 to 2016), and whether the outpatients were recruited consecutively. This information is important to ensure that no selection bias occurred in this study. Please clarify in the methods section, and if necessary, please discuss accordingly.

 5) In the initial PK study using non-compartmental analysis of the same participants (Schutz *et al.*, 2020 ^1^), the reviewer noticed that 1 of the 60 inpatients was excluded from the analysis due to alternative diagnosis of mycetoma and a high CD4 count. However, in the present study, it appears that all the 60 inpatients were included in the analysis. Are there any specific reasons why this one particular patient with an alternative diagnosis of mycetoma was included in the analysis of the present study?

 6) In Table 2, the reviewer noticed that the median (IQR) CD4 count in the outpatient group (n=48) was 142 (35.5-241) cells/mcL, whereas in the previous paper by Schutz *et al*., ^1^ the median (IQR) CD4 count in the outpatient group (n=48) was 146 (37-233) cells/mcL. Please clarify this difference, and please ensure that the baseline characteristic values presented in the present study are similar to those in the previous study by Schutz *et al* ^1^.

 7) The investigators found that, in general, the pharmacokinetics of first-line anti TB drugs are not significantly different between inpatients with HIV/TB and outpatients with or without HIV/TB. The reviewer wondered whether the difference in pharmacokinetics can be observed when comparing patients with advanced HIV/AIDS disease (WHO clinical stage 3 or 4) and those with an early stage of HIV infection (WHO clinical stage 1 or 2), irrespective of hospitalization status. The reviewer suggests that the authors perform a subgroup analysis for each drug in patients with HIV-associated TB by taking into account the severity of the HIV/AIDS disease.

 8) In this study, the authors did not find significant relationships between PK parameters of each drug and HIV status. As have been discussed by the authors in the manuscript, previous studies have produced contrasting results regarding these relationships. However, it is important to note that in a recent individual patient data meta-analysis in children with TB, HIV-positive status was significantly associated with lower plasma exposures to all first-line anti-TB drugs (see Gafar F, *et al*., 2023 ^2^).

 9) Finally, the reviewer believes that further discussion of the clinical implications of the results and also future perspectives would strengthen the study and be of interest to readers, not only from pharmaceutical but also from clinical backgrounds.

 10) Minor: in Table 3, ‘[43%]’ should be moved after ’93.8 (67.4-111)’?; in Table 2, is CD4 count data available for all outpatients or only for a subset of outpatients with HIV/TB?; change ‘rifmapicin’srifampicin’s’ in page 11 to ‘rifampicin’ – please clarify.

 I hope all my comments and suggestions will help the authors improve their manuscript.

Is the work clearly and accurately presented and does it cite the current literature?

Partly

If applicable, is the statistical analysis and its interpretation appropriate?

Yes

Are all the source data underlying the results available to ensure full reproducibility?

Yes

Is the study design appropriate and is the work technically sound?

Yes

Are the conclusions drawn adequately supported by the results?

Yes

Are sufficient details of methods and analysis provided to allow replication by others?

Yes

Reviewer Expertise:

Clinical pharmacology of anti-TB drugs, pharmacotherapy of TB, epidemiology of TB.

I confirm that I have read this submission and believe that I have an appropriate level of expertise to confirm that it is of an acceptable scientific standard, however I have significant reservations, as outlined above.

## References

[1] : Early antituberculosis drug exposure in hospitalized patients with human immunodeficiency virus-associated tuberculosis. *Br J Clin Pharmacol* .2020;86(5) : 10.1111/bcp.14207 966-978 10.1111/bcp.14207 31912537 PMC7163385

[2] : Global estimates and determinants of antituberculosis drug pharmacokinetics in children and adolescents: a systematic review and individual patient data meta-analysis. *Eur Respir J* .2023;61(3) : 10.1183/13993003.01596-2022 10.1183/13993003.01596-2022 36328357 PMC9996834

# Reviewer report: Reviewer response for version 2

**DOI:** 10.21956/wellcomeopenres.20580.r53462

**Published:** 2023-03-02

**Authors:** Oscar Della Pasqua

**Affiliations:** 1 Clinical Pharmacology & Therapeutics, School of Life and Medical Sciences, University College London, London, UK

I would like to thank the authors for their point-by-point reply to my previous queries. They have addressed all the points accordingly.

 The revised version has improved considerably.

Is the work clearly and accurately presented and does it cite the current literature?

Partly

If applicable, is the statistical analysis and its interpretation appropriate?

Yes

Are all the source data underlying the results available to ensure full reproducibility?

No

Is the study design appropriate and is the work technically sound?

Partly

Are the conclusions drawn adequately supported by the results?

Partly

Are sufficient details of methods and analysis provided to allow replication by others?

Partly

Reviewer Expertise:

Clinical pharmacology, modelling and simulation, tuberculosis, paediatrics, biomarkers, clinical drug development

I confirm that I have read this submission and believe that I have an appropriate level of expertise to confirm that it is of an acceptable scientific standard.

# Reviewer report: Reviewer response for version 1

**DOI:** 10.21956/wellcomeopenres.19537.r51522

**Published:** 2022-08-08

**Authors:** Jotam Pasipanodya

**Affiliations:** 1 Quantitative Preclinical & Clinical Sciences Department, Praedicare Inc., Dallas, TX, USA

The manuscript by Abdelgawad *et al.*, which compares pharmacokinetics (PK) of first-line anti-tuberculosis drugs in hospitalized patients with concurrent HIV to ambulatory patients, does not give any new meaningful information in its current form.

 Professors’ McIlleron, Wilkinson and Maartens groups has led the field of PK and pharmacodynamics (PD) of anti-TB drugs in patients with/out HIV. A careful review of past studies from the group could have improved the introduction and discussion, and therefore possibly sharpen the hypothesis of whether PK variability impacts early TB mortality. As an example, several studies from the group have identified low rifampin/pyrazinamide exposures and some of those studies, including that by Sekaggya-Wiltshire *et al., *(2018) ^1^ **revealed that those low exposures associated with slow culture conversion in TB/HIV patients. Therefore, this reader is surprised that the authors only sought to determine if PKs of hospitalized [inpatients] patients were different from outpatients. The group already have enough data to answer that question. However, the comparison of PKs of hospitalized deaths to survivors and outpatients [shown in Figure 1], is indeed an interesting question which was not fully explored by the authors. Importantly, a table of comparison of baseline characteristics, including both anti-TB and HAART drug doses, stratified by outpatients, hospitalized survivors and hospitalized deaths, would help readers. Vinnard *et al., *(2017) ^2^ has published PKs of anti-TB drugs in HIV patients, including examining effect of viraemia on rifampin absorption.

 Finally, drug-drug interactions in the PKs of TB/HIV patients must not only be qualitatively described, but rather investigated quantitatively. Concentration-dependent antagonism has been reported with anti-TB drugs in animal models: it would be interesting to see if those correlate with outcomes in TB/HIV.

Is the work clearly and accurately presented and does it cite the current literature?

No

If applicable, is the statistical analysis and its interpretation appropriate?

Yes

Are all the source data underlying the results available to ensure full reproducibility?

Yes

Is the study design appropriate and is the work technically sound?

Yes

Are the conclusions drawn adequately supported by the results?

Partly

Are sufficient details of methods and analysis provided to allow replication by others?

Yes

Reviewer Expertise:

Infectious diseases, pharmacometrics

I confirm that I have read this submission and believe that I have an appropriate level of expertise to confirm that it is of an acceptable scientific standard, however I have significant reservations, as outlined above.

## References

[1] : Delayed Sputum Culture Conversion in Tuberculosis–Human Immunodeficiency Virus–Coinfected Patients With Low Isoniazid and Rifampicin Concentrations. *Clinical Infectious Diseases* .2018;67(5) : 10.1093/cid/ciy179 708-716 10.1093/cid/ciy179 PMC609400329514175

[2] : Markers of gut dysfunction do not explain low rifampicin bioavailability in HIV-associated TB. *Journal of Antimicrobial Chemotherapy* .2017;72(7) : 10.1093/jac/dkx111 2020-2027 10.1093/jac/dkx111 PMC589069628472448

# Response

**Published:** 2022-11-02

**Authors:** Noha Abdelgawad

**Affiliations:** University of Cape, South Africa

We thank the reviewer for his comments and suggestions. We include below a point-by-point response to the comments, outlining the changes we made following the suggestions.

*“The manuscript by Abdelgawad et al., which compares pharmacokinetics (PK) of first-line anti-tuberculosis drugs in hospitalized patients with concurrent HIV to ambulatory patients, does not give any new meaningful information in its current form.*

*Professors’ McIlleron, Wilkinson and Maartens groups has led the field of PK and pharmacodynamics (PD) of anti-TB drugs in patients with/out HIV. A careful review of past studies from the group could have improved the introduction and discussion, and therefore possibly sharpen the hypothesis of whether PK variability impacts early TB mortality. As an example, several studies from the group have identified low rifampin/pyrazinamide exposures and some of those studies, including that by Sekaggya-Wiltshire et al., (2018)* *1* *revealed that those low exposures associated with slow culture conversion in TB/HIV patients. Therefore, this reader is surprised that the authors only sought to determine if PKs of hospitalized [inpatients] patients were different from outpatients. The group already have enough data to answer that question.”*

**Response**: In this paper, we report the pharmacokinetic analysis of rifampicin, isoniazid, and pyrazinamide within a pharmacokinetic study in hospitalized TB/HIV patients with TB patients with or without HIV enrolled as controls in South African patients. The paper addresses a knowledge gap due to the lack of previous publications comparing rifampicin, isoniazid, and pyrazinamide pharmacokinetics between hospitalized and non-hospitalized patients during early days of treatment. None of the previous studies, to our knowledge, looked at acutely ill patients on day 3 of TB treatment. Although multiple studies describe the pharmacokinetics of first-line TB drugs once the patients are established on treatment, there is a paucity of data looking at the pharmacokinetics during early treatment among patients hospitalized for acute illness, for whom the mortality rates are very high during the first 1 - 2 weeks of hospitalization.

 In this study, we investigate if there is a difference in the PK parameters and drug concentrations between hospitalized and non-hospitalized patients. Our report highlights that hospitalized TB patients’ mortality is not due to underexposure of antitubercular drugs. Although these patients may benefit from higher doses, exposure in hospitalized patients was similar to outpatients and not lower in inpatients who died. The report also introduces some new findings, particularly, the slower rifampicin absorption and higher between-subject variability in pyrazinamide clearance in hospitalized deaths compared to hospitalized survivors and in hospitalized vs outpatients. So, while rifampicin absorption is slower in hospitalized patients, the bioavailability is similar between hospitalized who died, hospitalized who survived and outpatients, which means that intravenous rifampicin administration may be not be necessary for hospitalized TB patients. A section was added to the discussion to further clarify this point.

 We did not have any detailed pharmacodynamic (PD) measures such as culture conversion times to explore PK-PD relationships, and the study was not powered to investigate PK-PD relationships. The only PD data collected was whether the participant died within 12-weeks of hospitalization or not. The patients hospitalized were critically ill patients, who usually die within the first week or two of hospitalization. In such a cohort, mortality is more likely due the severity of patient’s illness and their critical condition at presentation to hospital. The effect of anti-TB drugs concentrations will be very difficult to extricate from those other factors, especially if all patients receive similar doses and in such a small cohort. For the same reasons, the data in this study does not allow exploration of concentration-dependent antagonism. 

 We thank the reviewer for the chance to improve the manuscript. A section was added to the introduction to highlight that lower antitubercular drug concentrations have been shown to be associated with worsened sputum measures of mycobacterial load as shown by Sekaggya-Wiltshire et al. [1] and Mah et al. [2].

*“Finally, drug-drug interactions in the PKs of TB/HIV patients must not only be qualitatively described, but rather investigated quantitatively. Concentration-dependent antagonism has been reported with anti-TB drugs in animal models: it would be interesting to see if those correlate with outcomes in TB/HIV.”*

**Response**: Drug-drug interactions of first-line anti-TB drugs with antiretroviral drugs were not the main question of this analysis. The main objective was to see what the differences in the pharmacokinetics are between hospitalized (survivors and deaths) and outpatients. However, as part of the analysis, the effect of ART drugs (efavirenz, lopinavir/ritonavir, lamivudine) was tested on the different pharmacokinetic parameters of the 3 drugs and was not significant. Only 25/108 (23%) participants were on ARTs. Only 9 were on efavirenz, 9 on tenofovir, 1on lopinavir/ritonavir, and 1 on lamivudine. The concurrent ART therapy is now highlighted in the manuscript.

*“However, the comparison of PKs of hospitalized deaths to survivors and outpatients [shown in Figure 1], is indeed an interesting question which was not fully explored by the authors. Importantly, a table of comparison of baseline characteristics, including both anti-TB and HAART drug doses, stratified by outpatients, hospitalized survivors and hospitalized deaths, would help readers.”*

**Response**: Table 2 has been replaced with one stratified into outpatients, hospitalized survivors, hospitalized deaths instead of outpatients and hospitalized patients. Details regarding the HIV status, CD4 count, viral loads, and current antiretroviral (ART) therapy were added to the table as well. Thanks for this suggestion.

*“Vinnard et al., (2017)* *2* *has published PKs of anti-TB drugs in HIV patients, including examining effect of viraemia on rifampin absorption.”*

**Response**: Trefoil factor-3, a marker of gut barrier integrity, was tested as a covariate on the absorption and bioavailability of rifampicin but its effect was not found to be statistically significant. This is now clarified in the manuscript. This agrees with Vinnard *et al.* who quantified intestinal damage and microbial translocation by measuring levels of I-FABP and sCD14. They concluded that variability in markers of gut damage and microbial translocation did not contribute to variability in the oral bioavailability of rifampicin [3].

**References**:

 1. Sekaggya-Wiltshire C, Von Braun A, Lamorde M, Ledergerber B, Buzibye A, Henning L, et al. Delayed Sputum Culture Conversion in Tuberculosis–Human Immunodeficiency Virus–Coinfected Patients With Low Isoniazid and Rifampicin Concentrations. Clin Infect Dis [Internet]. Oxford University Press; 2018 [cited 2022 Oct 18];67:708–16. Available from: /pmc/articles/PMC6094003/

 2. Mah A, Kharrat H, Ahmed R, Gao Z, Der E, Hansen E, et al. Serum drug concentrations of INH and RMP predict 2-month sputum culture results in tuberculosis patients. Int J Tuberc Lung Dis. 2015;19:210–5.

 3. Vinnard C, Ravimohan S, Tamuhla N, Pasipanodya J, Srivastava S, Modongo C, et al. Markers of gut dysfunction do not explain low rifampicin bioavailability in HIV-associated TB.

# Reviewer report: Reviewer response for version 1

**DOI:** 10.21956/wellcomeopenres.19537.r48936

**Published:** 2022-06-06

**Authors:** Oscar Della Pasqua

**Affiliations:** 1 Clinical Pharmacology & Therapeutics, School of Life and Medical Sciences, University College London, London, UK

In this manuscript Abdelgawad and colleagues propose to compare the population pharmacokinetics of rifampicin, isoniazid, and pyrazinamide between hospitalized and non-hospitalized tuberculosis patients with or without HIV.

 I congratulate the authors for considering drug exposure as a potential determinant of the clinical deterioration and TB-associated complications in HIV+ patients. However, the manuscript has some important limitations, which need to be considered carefully to ensure accurate interpretation of the results. Given the heterogeneity of the patient population and available first and second line antiretroviral therapies for HIV, the conclusions drawn by the authors cannot be generalised.

 The most important limitation is the complete lack of details on the background therapy used for the treatment of HIV (i.e., which drugs, which doses and dosing regimens). The rationale for the current investigation is that hospitalised TB/HIV+ patients are more likely to have a poor prognosis. There are numerous publications highlighting that some, but not all, antiretroviral drugs show clinically relevant interaction with rifampicin. On the other hand, rifampicin is known to be a strong CYP3A4 inducer. For instance, both pre-systemic and systemic induction by efavirenz-based ART has been shown to affect rifampicin pharmacokinetics (See e.g., Sundell *et al*., 2021 ^1^). For the HIV-1 protease inhibitors (PIs) lopinavir, atazanavir (ATV), and darunavir, intestinal absorption and hepatic elimination are largely governed by CYP3A4/5 activity, and as such affected by co-administration of metabolic inducers such as rifampicin (See e.g., Montanha *et al*., 2022 ^2^).

 Apparently, the focus of the data analysis presented here was to assess whether significant differences exist in the pharmacokinetics of first line therapy drugs, and consequently differences in drug exposure in TB/HIV+ patients who are hospitalised, relative to those who do not require hospitalisation. Their analysis does not consider the possibility of a pharmacokinetic drug-drug interaction with anti-retrovirals.

 Therefore, to ensure appropriate interpretation of the results, further details should have been provided on the use of concurrent HIV co-medications. In addition, readers would benefit from an overview of the clinical and demographic baseline characteristics of the patients who died within 12 weeks from the start of the treatment with anti-tubercular drugs, including an outline of their viral load and CD4 counts, as compared to those who are not hospitalised or do not deteriorate during the course of therapy.

**Minor comments:**

 1. Study objectives. The authors state that the aim of the study was to compare the pharmacokinetics of rifampicin, isoniazid, and pyrazinamide between hospitalised patients and outpatients recruited from the same hospital catchment area. However this does not fully reflect the title of the manuscript. What is the relevance of HIV co-infection? There is no emphasis on the role of concurrent disease; the comparison seems to be limited to the patient condition (i.e., hospitalised vs. outpatients).

 2. In addition, the method section is not very clear. First, it is stated that the study population is made of two groups (hospitalised vs. outpatients (control group)). Then details are given about the inclusion of HIV+ and HIV – subjects to the outpatient cohort. There is no explanation why HIV+ and HIV- subjects were not included in the hospitalised group. Apparently, the hospitalised group consists only of TB/HIV+ patients.

 3. Inclusion criteria. The authors should have listed the cause of hospitalisation and explain why no specific antiretroviral therapy has been defined as inclusion/exclusion criteria. Restricting the inclusion of patients on regimens with high probability of drug-drug interaction would have made this investigation more relevant to clinicians and healthcare providers.

 4. First line therapy in drug susceptible tuberculosis consists of four drugs. The authors do not provide any explanation for the exclusion of ethambutol from this analysis. The authors should also highlight the potential implications of variable exposure to ethambutol.

 5. Assuming that the analysis focuses primarily on the effect of hospitalisation on the pharmacokinetic properties of anti-tubercular drugs, justification is missing for the sample size of the groups. Given the role of demographic baseline characteristics on the pharmacokinetic disposition of both antiretroviral and antitubercular drugs, it would be important to understand which parameters will be of primary interest during the data analysis.

 6. There are many publications on the population pharmacokinetics of antitubercular drugs (e.g., Muliaditan *et al.*, 2019 ^3^; Muda *et al*., 2022 ^4^), including investigations where findings contrast with results and conclusions drawn by the authors (Sundell *et al*., 2021 ^5^; Rao *et al.*, 2021 ^6^). Unfortunately, these references were not included or considered for discussion. Consequently, the work is presented as if this was the first time population pharmacokinetic models were being developed. Prior distributions and structural model(s) could have been used for the purpose of the current analysis. This would have provided an opportunity to optimise sampling times and focus on features such as absorption rate. Similarly, the use of priors describing parameter distributions would have allowed the authors to assess the pharmacokinetics of anti-retroviral drugs.

 7. Covariate model building. The authors do not explain the lack of a time varying clearance for rifampicin, as autoinduction is a well-known process in the elimination of this drug. Under autoinduction conditions, the elimination half-life of rifampicin decreases significant, as clearance increases. It seems that the authors have disregarded these mechanisms and focused on the assumption of stochastic processes to explain variability, making the analysis of the data descriptive, rather than mechanistic or mechanism-based.

 8. Interestingly, the model includes between-occasion variability but relies on fixed point estimates for the bioavailability. This should be better explained and justified. Fixing a parameter suggests that no reference group was considered. By using interoccasion variability, differences in clearance (i.e., metabolic clearance) due to specific processes. This would have provided a more biologically plausible description of the underlying metabolic autoinduction.

 9. Mean transit time, absorption rate constants. The authors should present a more detailed outline of the rationale for the use of transit compartments. It is unclear whether the use of this approach was due to intrinsic drug absorption properties or due to extrinsic factors (e.g., formulation, food effect).

 10. This analysis would benefit from a summary including secondary pharmacokinetic parameters (i.e., post-hoc estimates of the individual curves and subsequent calculation of AUC, C _max_, C _ss_, C _min_). These parameters are shown graphically in Figure 3, but were not summarised numerically. Readers will find it much easier to compare and interpret these results.

 11. It has been previously established that there a rise in serum bilirubin during treatment with rifampicin. This is due to the competition between rifampicin and bilirubin for hepatic uptake and excretion. However, this is relevant primarily in individuals who have hepatic impairment or other comorbidities such as cirrhosis. The authors need to explain the inclusion criteria and cause of hospitalisation, as the normal range for conjugated bilirubin is < 5.1 μmol/L. The values depicted in figure 2 should not be applicable to HIV or TB patients who do not experience hepatic toxicity or have other co-morbidities (i.e., hepatic disease).

 12. There is no explanation why interindividual variability was not identified for the volume of distribution. There seems to be a sufficiently large interindividual variation in body weight and fat free mass. In addition, the authors did not report the  shrinkage for ETA on clearance.

 13. The discussion should present further details on the limitations outlined in the previous points. In particular, the authors need to make clear that their results cannot be generalised given the lack of details on the inclusion criteria and background antiretroviral therapy of the patients included in this analysis.

 14. I would recommend that the authors share the NONMEM control stream files as supplementary material.

Is the work clearly and accurately presented and does it cite the current literature?

Partly

If applicable, is the statistical analysis and its interpretation appropriate?

Yes

Are all the source data underlying the results available to ensure full reproducibility?

No

Is the study design appropriate and is the work technically sound?

Partly

Are the conclusions drawn adequately supported by the results?

Partly

Are sufficient details of methods and analysis provided to allow replication by others?

Partly

Reviewer Expertise:

Clinical pharmacology, modelling and simulation, tuberculosis, paediatrics, biomarkers, clinical drug development

I confirm that I have read this submission and believe that I have an appropriate level of expertise to confirm that it is of an acceptable scientific standard, however I have significant reservations, as outlined above.

## References

[1] : Effect of efavirenz-based ART on the pharmacokinetics of rifampicin and its primary metabolite in patients coinfected with TB and HIV. *Journal of Antimicrobial Chemotherapy* .2021;76(11) : 10.1093/jac/dkab258 2950-2957 10.1093/jac/dkab258 PMC852140334337654

[2] : Predicting Drug-Drug Interactions between Rifampicin and Ritonavir-Boosted Atazanavir Using PBPK Modelling. *Clin Pharmacokinet* .61(3) : 10.1007/s40262-021-01067-1 375-386 10.1007/s40262-021-01067-1 34635995 PMC9481493

[3] : How long will treatment guidelines for TB continue to overlook variability in drug exposure?. *Journal of Antimicrobial Chemotherapy* .2019;74(11) : 10.1093/jac/dkz319 3274-3280 10.1093/jac/dkz319 PMC796782931360999

[4] : Population pharmacokinetics analyses of rifampicin in adult and children populations: A systematic review. *Br J Clin Pharmacol* .2022; 10.1111/bcp.15298 10.1111/bcp.15298 35253251

[5] : Factors Affecting the Pharmacokinetics of Pyrazinamide and Its Metabolites in Patients Coinfected with HIV and Implications for Individualized Dosing. *Antimicrobial Agents and Chemotherapy* .2021;65(7) : 10.1128/AAC.00046-21 10.1128/AAC.00046-21 PMC837325133875424

[6] : Population Pharmacokinetics and Significant Under-Dosing of Anti-Tuberculosis Medications in People with HIV and Critical Illness. *Antibiotics (Basel)* .2021;10(6) : 10.3390/antibiotics10060739 10.3390/antibiotics10060739 34207312 PMC8235594

# Response

**Published:** 2022-11-02

**Authors:** Noha Abdelgawad

**Affiliations:** University of Cape, South Africa

We thank the reviewer for the useful input on our manuscript. We include below a point-by-point response to the comments. The changes we made following the suggestions are outlined.

*“In this manuscript Abdelgawad and colleagues propose to compare the population pharmacokinetics of rifampicin, isoniazid, and pyrazinamide between hospitalized and non-hospitalized tuberculosis patients with or without HIV.*

*I congratulate the authors for considering drug exposure as a potential determinant of the clinical deterioration and TB-associated complications in HIV+ patients. However, the manuscript has some important limitations, which need to be considered carefully to ensure accurate interpretation of the results. Given the heterogeneity of the patient population and available first and second line antiretroviral therapies for HIV, the conclusions drawn by the authors cannot be generalised.*

*The most important limitation is the complete lack of details on the background therapy used for the treatment of HIV (i.e., which drugs, which doses and dosing regimens).* *The rationale for the current investigation is that hospitalised TB/HIV+ patients are more likely to have a poor prognosis. There are numerous publications highlighting that some, but not all, antiretroviral drugs show clinically relevant interaction with rifampicin. On the other hand, rifampicin is known to be a strong CYP3A4 inducer. For instance, both pre-systemic and systemic induction by efavirenz-based ART has been shown to affect rifampicin pharmacokinetics (See e.g., Sundell et al., 2021* *1* *). For the HIV-1 protease inhibitors (PIs) lopinavir, atazanavir (ATV), and darunavir, intestinal absorption and hepatic elimination are largely governed by CYP3A4/5 activity, and as such affected by co-administration of metabolic inducers such as rifampicin (See e.g., Montanha et al., 2022* *2* *).*

*Apparently, the focus of the data analysis presented here was to assess whether significant differences exist in the pharmacokinetics of first line therapy drugs, and consequently differences in drug exposure in TB/HIV+ patients who are hospitalised, relative to those who do not require hospitalisation. Their analysis does not consider the possibility of a pharmacokinetic drug-drug interaction with anti-retrovirals.”*

**Response:** Indeed, the focus of this manuscript is comparing the pharmacokinetics of rifampicin, isoniazid, and pyrazinamide between tuberculosis patients in need of hospitalization vs tuberculosis outpatients. The objectives of this study did not specifically include exploring the pharmacokinetics of anti-retrovirals or drug-drug interactions between antitubercular and antiretroviral drugs. The concentrations of antiretroviral drugs were not quantified, so we cannot explore the effect of the antitubercular drugs on their pharmacokinetics. Details regarding the concomitant HIV drugs used are now added to the manuscript.

 We agree that the concomitant use of efavirenz with rifampicin is expected to have an effect on rifampicin’s pharmacokinetics, as shown by *Sundell et al.* ^1^. However, when we tested efavirenz as a covariate on rifampicin PK parameters, there was no significant effect. We think this could be due to a number of reasons. First, possible non-compliance of patients to their ART regimens prior to hospitalisation (since efavirenz plasma levels were not quantified, it is not possible to ascertain whether they took their efavirenz doses or not). Secondly, *Sundell et al*. ^1^ looked at rifampicin PK on the first day of treatment, when no rifampicin autoinduction is present and the difference between patients taking efavirenz or not is expected to be maximal. In our study, rifampicin PK was observed on day 3, when some of rifampicin’s autoinduction has already happened, thus possibly reducing the expected difference between patients taking efavirenz or not. Consistently with this hypothesis, several previous studies looking into rifampicin-efavirenz interaction found no effect at steady-state, when rifampicin auto-induction is complete ^2,3^.

 Table 1 is now stratified into outpatients, hospitalized survivors, hospitalized deaths instead of outpatients and hospitalized patients. Details regarding the HIV status, CD4 count, and concurrent HIV comedications have been added to the table as well. Thanks for the suggestion.

*Minor comments:*

*1. Study objectives. The authors state that the aim of the study was to compare the pharmacokinetics of rifampicin, isoniazid, and pyrazinamide between hospitalised patients and outpatients recruited from the same hospital catchment area. However, this does not fully reflect the title of the manuscript. What is the relevance of HIV co-infection? There is no emphasis on the role of concurrent disease; the comparison seems to be limited to the patient condition (i.e., hospitalised vs. outpatients).*

**Response:** The title was changed to “Pharmacokinetics of antitubercular drugs in patients hospitalized with HIV-associated tuberculosis: a population modelling analysis” to better reflect the study objectives.

*2. In addition, the method section is not very clear. First, it is stated that the study population is made of two groups (hospitalised vs. outpatients (control group)). Then details are given about the inclusion of HIV+ and HIV – subjects to the outpatient cohort. There is no explanation why HIV+ and HIV- subjects were not included in the hospitalised group. Apparently, the hospitalised group consists only of TB/HIV+ patients.*

**Response:** HIV+ patients that were newly diagnosed with tuberculosis requiring hospitalization were enrolled as part of a large observational cohort study and the PK sub-study was performed in selected patients on the 3 ^rd^ day of treatment. The outpatient controls were enrolled after the completion of the hospital cohort study and for logistical reasons, both HIV+ and HIV- were included in the outpatients group. The focus of the PK sub-study was the comparison of antitubercular drug concentrations at a very early time point (the 3 ^rd^ day of treatment). Thus, for the outpatients control group, the inclusion criteria were not restricted to HIV+ patients only but rather to adults newly diagnosed with tuberculosis who can be enrolled prior to receiving their 3 ^rd^ dose of TB therapy.

*3. Inclusion criteria. The authors should have listed the cause of hospitalisation and explain why no specific antiretroviral therapy has been defined as inclusion/exclusion criteria. Restricting the inclusion of patients on regimens with high probability of drug-drug interaction would have made this investigation more relevant to clinicians and healthcare providers.*

**Response: **Thanks for giving us the chance to clarify this point in the manuscript. The main focus of the PK study was to compare the pharmacokinetics of rifampicin, isoniazid and pyrazinamide in patients with different illness severity i.e., patients who presented to care in need of hospitalization due to tuberculosis (who were too ill to receive initial treatment as outpatients) vs those with tuberculosis that do not require hospitalization (the outpatients control group). The study was not designed to explore the drug-drug interactions of the first-line antitubercular drugs with anti-retrovirals. Therefore, patients were enrolled regardless of their ART therapy status. At the time of study design, the publications showing possible drug-drug interactions such as *Sundell et al.* ^1^ with anti-retrovirals had not been published yet.

*4. First line therapy in drug susceptible tuberculosis consists of four drugs. The authors do not provide any explanation for the exclusion of ethambutol from this analysis. The authors should also highlight the potential implications of variable exposure to ethambutol.*

**Response:** We thank the reviewer for pointing this out and reminding us to explain this more in detail. In this study, we decided to focus on the PK of rifampicin, isoniazid, and pyrazinamide, because they are reported to be more effective and faster-acting during the early days of TB treatment. So, these drugs are thought to be more important for the hospitalised patients in the study, who usually die within the first few weeks of hospitalization. Unlike the other three drugs, ethambutol’s standard plasma-based pharmacokinetic-pharmacodynamic profile suggests that it may be of limited clinical value. Ethambutol is a bacteriostatic drug that lacks sterilizing activity and that should not be used alone for TB treatment because it has limited potency against slow-growing and nonreplicating bacteria ^4^. Its inclusion in the TB regimens is mainly justified because of its protective role against rifampicin resistance development. Therefore, due to limited funding available we preferred to analyze the three bactericidal drugs in a larger number of patients, rather than to measure all four drugs in fewer patients. The exclusion of ethambutol from the analysis was added as a limitation to the discussion section.

*5. Assuming that the analysis focuses primarily on the effect of hospitalisation on the pharmacokinetic properties of anti-tubercular drugs, justification is missing for the sample size of the groups. Given the role of demographic baseline characteristics on the pharmacokinetic disposition of both antiretroviral and antitubercular drugs, it would be important to understand which parameters will be of primary interest during the data analysis.*

**Response:** The analysis focuses on the differences between patients with HIV-associated tuberculosis who are ill enough to be hospitalized at the time of TB diagnosis and ambulant outpatients. The hospitalized group has an extremely high mortality rate and the rationale of the study is that the severity of their illness may lead to underexposure of antitubercular drugs which may contribute to their mortality. Sample size calculation was done for the main prospective observational cohort study to investigate factors associated with mortality among hospitalized patients with tuberculosis, whose primary outcome was dichotomized into survivor vs fatal outcome factoring in a cumulative mortality of 15%. However, since the PK study was exploratory, no formal sample size calculation was made.

*6. There are many publications on the population pharmacokinetics of antitubercular drugs (e.g., Muliaditan* *et al.* *, 2019* *3* *; Muda* *et al* *., 2022* *4* *), including investigations where findings contrast with results and conclusions drawn by the authors (Sundell* *et al* *., 2021* *5* *; Rao* *et al.* *, 2021* *6* *). Unfortunately, these references were not included or considered for discussion. Consequently, the work is presented as if this was the first time population pharmacokinetic models were being developed. Prior distributions and structural model(s) could have been used for the purpose of the current analysis. This would have provided an opportunity to optimise sampling times and focus on features such as absorption rate. Similarly, the use of priors describing parameter distributions would have allowed the authors to assess the pharmacokinetics of anti-retroviral drugs.*

**Response:** Thank you for the chance to enrich the discussion section. As the reviewer mentions, there are indeed many publications describing the popPK of these drugs, but large differences are sometimes reported between studies, possibly due to issues such as drug formulations, drug assays, etc. For this reason, it would have not been straightforward to choose the priors to use in the analysis and therefore make an “external” comparison. Rather, we recruit an “ad hoc” control group of patients from the same area, sampled at the same time after treatment initiation, and receiving the same drug formulations. The collected data was enough to support estimation of parameters without using priors and allow an “internal” comparison between the hospitalised and non-hospitalised patients, which we believe provides stronger results than using priors or historical data as a comparison. However, we agree that the discussion should be extended to and some of the references the reviewer suggested had not been published at the time of writing the manuscript. The discussion section has now been expanded to elaborate more on previous publications.

 We have decided not to include the models by Rao *et al.* ^5^ in our discussion, because their findings are very odd to reconcile with all previous literature. They report pharmacokinetics in patients with sepsis from TB bloodstream infection or meningitis after 2 weeks of treatment. Their models are one-compartment for rifampicin and pyrazinamide and two-compartments for isoniazid, which is in line with ours. However, their rifampicin clearance parameter estimate of 0.1 L/h is puzzling and unlike any previously published models, as summarized in the review by Muda *et al.* ^6^. For their isoniazid model, they included mid-upper-arm circumference (MUAC) on central volume, but MUAC has not been proven to be an accurate body size descriptor. Allometric scaling using descriptors that take into account both body size and composition (e.g. fat-free mass) are the gold standard and have been established as more accurate predictors for differences in pharmacokinetics ^7^. Moreover, they conclude that the patients were underexposed to the anti-TB drugs due to their critical illness, but no comparison was done with a control group (outpatients). In our study, exposures were often also below the generally accepted targets, but importantly the hospitalized patients were not underexposed compared to the outpatients control group.

 As for the pyrazinamide model by Sundell *et al.* ^8^, they found relative bioavailability to be ~50% higher in females compared to males, but they used no allometry to adjust for body size. We found fat-free mass to be better than total body weight when included in the model using allometric scaling in capturing the effect of body size on differences in apparent clearance and volume. Allometric scaling by fat-free mass ascribes PK differences to changes in body size and composition. It is more physiologically plausible and more parsimonious than using sex as a categorical covariate. It is also a well-established concept ^7^. This is also supported by findings from Muliaditan *et al.* ^9^, Rockwood *et al.* ^10^, and McIlleron *et al.* ^3^ who have shown that patients with lower body weights are underexposed relative to patients with higher body weight. Since fat-free mass is the best body size descriptor for clearance and volume, but dosing is determined based on total weight, patients with lower weights end up being underexposed. Therefore, we agree that the clinical recommendation for the use of weight-banded dosing regimens should be reconsidered to account for the variability in body size and composition. This point has been elaborated in the discussion section.

 Additionally, Sundell *et al.* ^8^ report a significant effect for efavirenz co-administration on the volume of distribution, but there is no mechanistic basis for this interaction. Other studies specifically looking at HIV e.g., Rockwood *et al.* ^10^ have looked into the effect of efavirenz on pyrazinamide PK and found no significant effect.

 Regarding the ART comment, we agree that using prior models we could have detected large differences in PK, but we have to reiterate that the objective of the main observational study was to investigate factors relating to the high mortality of hospitalized tuberculosis patients and the PK sub-study was conducted to only compare the pharmacokinetics of antitubercular drugs in TB patients who are critically ill in need of hospitalization vs TB outpatients, during the early days of treatment. The concentrations of the anti-retroviral drugs were not assayed as their pharmacokinetics was not the objective of this investigation.

*7. Covariate model building. The authors do not explain the lack of a time varying clearance for rifampicin, as autoinduction is a well-known process in the elimination of this drug. Under autoinduction conditions, the elimination half-life of rifampicin decreases significant, as clearance increases. It seems that the authors have disregarded these mechanisms and focused on the assumption of stochastic processes to explain variability, making the analysis of the data descriptive, rather than mechanistic or mechanism-based.*

**Response:** The inclusion of auto-induction of clearance in the model was tested but did not improve the model fit, which could be due to the fact that all participants were sampled on the same day (the 3 ^rd^ day of treatment). This is now clarified in the results section.

*8. Interestingly, the model includes between-occasion variability but relies on fixed point estimates for the bioavailability. This should be better explained and justified. Fixing a parameter suggests that no reference group was considered. By using inter-occasion variability, differences in clearance (i.e., metabolic clearance) due to specific processes. This would have provided a more biologically plausible description of the underlying metabolic autoinduction.*

**Response:** As illustrated by Lavielle & Aarons ^11^, bioavailability cannot be identified in such a cohort since all participants were administered oral drugs and none intravenously, therefore the population value for bioavailability was fixed to 1 but was allowed to be different between the occasions. The occasions in this model are occasion 1 for day 2 (for the pre-dose sample) and occasion 2 for Day 3 of treatment. Significant day-to-day changes are expected in absorption parameters (absorption rate constant, bioavailability) ^12^. While rifampicin’s autoinduction is expected to have an effect on clearance, only one sample was available from the day before the day of the PK sampling (pre-dose sample), plus the time of the dose may not have been accurately reported for some of the patients. For these reasons, it is unlikely to be able to detect day-to-day changes in clearance. Moreover, the majority of pre-dose samples were below the limit of quantification for rifampicin.

*9. Mean transit time, absorption rate constants. The authors should present a more detailed outline of the rationale for the use of transit compartments. It is unclear whether the use of this approach was due to intrinsic drug absorption properties or due to extrinsic factors (e.g., formulation, food effect).*

**Response:** Thanks for giving the chance to clarify. For all three drugs, a delay in absorption was observed in the profiles. The use of the flexible transit compartment model describes the delay in absorption better than the standard absorption model (in terms of drop in the objective function and the visual predictive check) because it is more physiologically plausible as shown by Savic *et al.* ^13^. The drop in OFV with the transit model (df = 1) was 9.22 points for rifampicin, 107 points for isoniazid, and 23.7 points for pyrazinamide. All participants were fasted overnight, so there is no food effect. And no significant formulation effect was found, only a trend (only 2 participants had a different formulation (individual tablets instead of the fixed-dose combination tablets).

*10. This analysis would benefit from a summary including secondary pharmacokinetic parameters (i.e., post-hoc estimates of the individual curves and subsequent calculation of AUC, C _max_, C _ss_, C _min_). These parameters are shown graphically in Figure 3, but were not summarised numerically. Readers will find it much easier to compare and interpret these results.*

**Response:** Thank you for the suggestion. The AUC _0-24h_ and C _max_ values shown graphically in Figure 3 have now been added in a table (Table 4). 

*11. It has been previously established that there a rise in serum bilirubin during treatment with rifampicin. This is due to the competition between rifampicin and bilirubin for hepatic uptake and excretion. However, this is relevant primarily in individuals who have hepatic impairment or other comorbidities such as cirrhosis. The authors need to explain the inclusion criteria and cause of hospitalisation, as the normal range for conjugated bilirubin is < 5.1 μmol/L. The values depicted in figure 2 should not be applicable to HIV or TB patients who do not experience hepatic toxicity or have other co-morbidities (i.e., hepatic disease).*

**Response:** The main inclusion criteria for the hospitalized group was patients presenting to care in need of hospitalization due to tuberculosis and who are too ill to receive initial treatment as outpatients. These patients were hospitalized due to TB infection and many of these patients would have had disseminated TB with liver involvement, which means they had abnormal liver function tests including elevated bilirubin levels at the time of hospitalization. The bilirubin values presented in Table 2 and in Figure 2 are values measured during screening, i.e., before the start of treatment, which means that the high bilirubin levels were not due to rifampicin treatment.

*12. There is no explanation why interindividual variability was not identified for the volume of distribution. There seems to be a sufficiently large interindividual variation in body weight and fat free mass* *. In addition, the authors did not report the shrinkage for ETA on clearance.*

**Response:** All three models included allometric scaling of the volume of distribution by fat-free mass as a way to explain the differences between the participants with different body sizes. The values for interindividual variability in the volume of distribution were very small (2.5% for rifampicin) and its inclusion did not result in a better fit of the model (dOFV = -0.34, df = 1), therefore, it was not included. The shrinkage for the ETAs for the three drugs has now been added to Table 3.

*13. The discussion should present further details on the limitations outlined in the previous points. In particular, the authors need to make clear that their results cannot be generalised given the lack of details on the inclusion criteria and background antiretroviral therapy of the patients included in this analysis.*

**Response:** Thank you for raising this point. The limitations are now better mentioned in the discussion. The models cannot be extended to TB patients who are hospitalized for other causes such as renal failure, liver failure, which could significantly alter the antitubercular drugs pharmacokinetics. The study design did not allow us to characterize the possible drug-drug interactions between antitubercular and antiretroviral drugs. However, our study cohort is representative of the general South African population who gets hospitalized for tuberculosis, and the study was conducted on the 3 ^rd^ day of treatment since those hospitalized patients are at a substantial risk of death within 7 days of admission.

*14. I would recommend that the authors share the NONMEM control stream files as supplementary material.*

**Response:** Thank you for the recommendation. The NONMEM code is now available on GitHub at http://github.com/noha-abdelgawad/PMX-TB-Models/tree/main/Project.KDHTB.Hospitalized.

**References:**

 1. Sundell J, Bienvenu E, Äbelö A, Ashton M. Effect of efavirenz-based ART on the pharmacokinetics of rifampicin and its primary metabolite in patients coinfected with TB and HIV. *J Antimicrob Chemother* 2021; **76**: 2950–7. Available at: https://academic.oup.com/jac/article/76/11/2950/6333768. Accessed October 18, 2022.

 2. Bhatt NB, Barau C, Amin A, *et al.* Pharmacokinetics of Rifampin and Isoniazid in Tuberculosis-HIV-Coinfected Patients Receiving Nevirapine- or Efavirenz-Based Antiretroviral Treatment. *Antimicrob Agents Chemother* 2014; **58**: 3182–90. Available at: http://aac.asm.org/. Accessed February 8, 2021.

 3. McIlleron H, Rustomje R, Vahedi M, *et al.* Reduced antituberculosis drug concentrations in HIV-infected patients who are men or have low weight: Implications for international dosing guidelines. *Antimicrob Agents Chemother* 2012; **56**: 3232–8. Available at: /pmc/articles/PMC3370773/. Accessed February 28, 2021.

 4. Zimmerman M, Lestner J, Prideaux B, *et al.* Ethambutol Partitioning in Tuberculous Pulmonary Lesions Explains Its Clinical Efficacy. *Antimicrob Agents Chemother* 2017; **61**. Available at: /pmc/articles/PMC5571334/. Accessed October 17, 2022.

 5. Rao PS, Moore CC, Mbonde AA, *et al.* Population Pharmacokinetics and Significant Under-Dosing of Anti-Tuberculosis Medications in People with HIV and Critical Illness. *Antibiot 2021, Vol 10, Page 739* 2021; **10**: 739. Available at: https://www.mdpi.com/2079-6382/10/6/739/htm. Accessed October 14, 2022.

 6. Muda MR, Harun SN, Syed Sulaiman SA, *et al.* Population pharmacokinetics analyses of rifampicin in adult and children populations: A systematic review. *Br J Clin Pharmacol* 2022; **88**: 3132–52. Available at: https://bpspubs.onlinelibrary.wiley.com/doi/10.1111/bcp.15298.

 7. Anderson BJ, Holford NHG. Mechanism-based concepts of size and maturity in pharmacokinetics. *Annu Rev Pharmacol Toxicol* 2008; **48**: 303–32.

 8. Sundell J, Wijk M, Bienvenu E, Äbelö A, Hoffmann K-J, Ashton M. Factors Affecting the Pharmacokinetics of Pyrazinamide and Its Metabolites in Patients Coinfected with HIV and Implications for Individualized Dosing. *Antimicrob Agents Chemother* 2021; **65**. Available at: https://journals.asm.org/doi/10.1128/AAC.00046-21. Accessed October 14, 2022.

 9. Muliaditan M, Pasqua O Della. How long will treatment guidelines for TB continue to overlook variability in drug exposure?

 10. Rockwood N, Meintjes G, Chirehwa M, Wiesner L, Mcilleron H, Wilkinson RJ. HIV-1 Coinfection Does Not Reduce Exposure to Rifampin, Isoniazid, and Pyrazinamide in South African Tuberculosis Outpatients. 2016; **60**: 6050–9.

 11. Lavielle M, Aarons L. What do we mean by identifiability in mixed effects models? *J Pharmacokinet Pharmacodyn* 2016; **43**: 111–22.

 12. Mould DR, Upton RN. Basic concepts in population modeling, simulation, and model-based drug development - Part 2: Introduction to pharmacokinetic modeling methods. *CPT Pharmacometrics Syst Pharmacol* 2013; **2**.

 13. Savic RM, Jonker DM, Kerbusch T, Karlsson MO. Implementation of a transit compartment model for describing drug absorption in pharmacokinetic studies. *J Pharmacokinet Pharmacodyn* 2007; **34**: 711–26.
